# Formation of Neurointerfaces Based on Electrically Conductive Biopolymers by Two-Photon Polymerization Method

**DOI:** 10.3390/polym17101300

**Published:** 2025-05-09

**Authors:** Mikhail S. Savelyev, Artem V. Kuksin, Denis T. Murashko, Ekaterina P. Otsupko, Victoria V. Suchkova, Kristina D. Popovich, Pavel N. Vasilevsky, Yulia O. Vasilevskaya, Ulyana E. Kurilova, Elena M. Eganova, Polina A. Edelbekova, Sergey V. Selishchev, Alexander A. Pavlov, Alexander Yu. Gerasimenko

**Affiliations:** 1 Institute of Biomedical Systems, National Research University of Electronic Technology, 124498 Zelenograd, Russia; 8140241@edu.miet.ru (A.V.K.); 8191388@edu.miet.ru (E.P.O.); suchkova_v_v@staff.sechenov.ru (V.V.S.); popovich_k@staff.sechenov.ru (K.D.P.); u130020@edu.miet.ru (P.N.V.); kurilova_u_e@staff.sechenov.ru (U.E.K.); selishchev@bms.zone (S.V.S.); gerasimenko@bms.zone (A.Y.G.); 2 Institute for Bionic Technologies and Engineering, I. M. Sechenov First Moscow State Medical University, 119991 Moscow, Russia; 3 Scientific-Manufacturing Complex “Technological Centre”, 124498 Moscow, Russia; jo.fedorova@tcen.ru; 4 Institute of Nanotechnology of Microelectronics of the Russian Academy of Sciences, 119991 Moscow, Russia; eganova.e@inme-ras.ru (E.M.E.); edelbekova.p@inme-ras.ru (P.A.E.); pavlov.a@inme-ras.ru (A.A.P.)

**Keywords:** nonlinear optical properties, two-photon polymerization, chitosan, carbon nanotubes, eosin Y, neuronal engineering

## Abstract

Preventing false signals of phantom pain after limb amputation is crucial. The development of neurointerfaces capable of bidirectional information exchange between the brain and external devices, along with long-term use, is a key research priority. The main problem with existing devices lies in the potential formation of scar tissue and the death of adjacent neurons. To address this issue, a polymer composite based on new composition: chitosan, bovine serum albumin, single-walled carbon nanotubes, and Eosin Y, which was created for the fabrication of a neurointerface. A polymer composite of the required shape was formed by two-photon polymerization. In studying its nonlinear optical properties, the new effect of phase self-modulation was discovered, which is observed after exposure to laser radiation prior to the formation of the composite. The time of appearance of diffraction rings was measured. This allowed optimization of laser parameters—scanner speed and intensity. The resulting homogeneous composite exhibited a specific conductivity of 20 mS × cm^−1^, sufficient for electrophysiological signal transmission.

## 1. Introduction

In our previous work [1], the key achievement was the development of a hydrogel formulation capable of forming a composite under low-energy nanosecond infrared laser irradiation. The original composition used gelatin, whose properties exhibit significant sensitivity to minor temperature fluctuations. This temperature dependence substantially complicates shape control of the resulting material due to deformation occurring even during polymerization. To address this issue, we propose replacing gelatin with chitosan (CS). Ensuring polymerization stability is a critical challenge in developing neurointerface technology with high reproducibility.

The development of implantable devices for pain management has gained significant importance. The prevailing treatment modality entails the utilization of medications that necessitate continuous administration. Given the inherent variability among individuals, the efficacy of such treatment depends not only on the selection of medications, but also on the individual’s response. The phenomenon of phantom limb pain following amputation has been shown to be amenable to management through bidirectional neurointerfaces [2]. Neurointerfaces are critical components in the field of neural engineering, and their development hinges on the exploration of novel materials and their structuring. The objective is to engineer materials that exhibit a combination of properties, including biocompatibility [3], electrical conductivity [4,5], and the capacity to guide neurite growth [6,7], while ensuring strong nerve cells adhesion [8,9]. This approach is pivotal in preventing scar formation, which can impede the transmission and reception of control signals. To achieve this, it proposes designing a polymeric construction with a specific geometry, tailored to the site of implantation site. Biopolymer-based composites have shown the greatest promise in meeting these requirements [10,11]. Their properties are strongly influenced by the internal structure [4,12,13]. Localization is performed during the creation of composites by photopolymerization, enabling the formation of objects with various shapes without affecting the areas outside the laser exposure zone. It is essential to precisely control the energy parameters of the laser radiation used and the properties of the initial gel, which depend on its composition.

CS is a natural biopolymer, similar to cellulose in structure but differing by the presence of amino groups (-NH_2_) rather than hydroxyl groups (-OH) [11,14]. CS is made by removing the acetyl groups from chitin, and it can be produced in large quantities (mainly from the waste from shrimp and crab shells). At the same time, it is a biocompatible non-toxic cationic polysaccharide that supports nerve cell proliferation and differentiation [15]. CS exhibits extremely low electrical conductivity (<10^−7^ S×cm^−1^), making it unsuitable for bioelectronic applications requiring higher conductivity. To achieve the required conductivity (>1.0 mS×cm⁻¹) for electrical stimulation applications—where physiological potentials are typically limited to ~100 mV and biologically active currents range from 0.6 to 400 μA—carbon nanotubes are incorporated into the material [16,17]. This composite maintains adequate biocompatibility despite steric constraints caused by nanotube incorporation [8,18,19]. Such a modified biopolymer can serve as an effective neurointerface for direct interaction with nerve cells. The material also includes bovine serum albumin (BSA) to enhance biocompatibility, permitting higher concentrations of single-walled carbon nanotubes (SWCNTs) [20,21]. In the present work, Eosin Y dye from among type II photoinitiators is selected to provide photopolymerization. This FDA-approved dye for hematology has an absorption maximum of around 525 nm and 250–360 nm [22,23]. The maximum non-toxic concentration of Eosin Y is 0.25 mM [24]. The cytocompatibility of Eosin Y has been confirmed in many studies [25,26,27,28]. These properties make it suitable for visible-light and two-photon polymerization, a key requirement for this study.

These characteristics may indicate self-focusing or defocusing effects that could influence beam diameter. They may also reveal scattering phenomena potentially increasing absorption [29] through optical path elongation in the material. The nonlinear absorption cross-section was measured to validate the hydrogel bio-ink formulation. These bio-inks undergo polymerization by a 1070 nm wavelength laser of the low-energy IR range. Sample shaping is controlled through laser spot power and velocity modulation. This study focuses on phase self-modulation, a phenomenon enabling refractive index determination [30] and identification of polymerization onset and polymerization threshold.

For evaluating the biocompatibility of the nanocomposite, the mouse neuroblastoma cell line Neuro 2A was selected. The key advantages of this cell line include a rapid doubling time and its ability to differentiate into neurons within a few days [31]. Neuro 2A cells are widely used in studies on neuronal proliferation and differentiation [32,33], signaling pathways [34], neurite outgrowth [35], and cytotoxicity [36]. Additionally, they serve as a model cell line in electrophysiological research exploring the effects of electrical stimulation on nervous system regeneration [37,38,39].

## 2. Materials and Methods

### 2.1. Materials and Preparation of CS/BSA/Eosin Y/SWCNTs Composite

**Figure 1:**
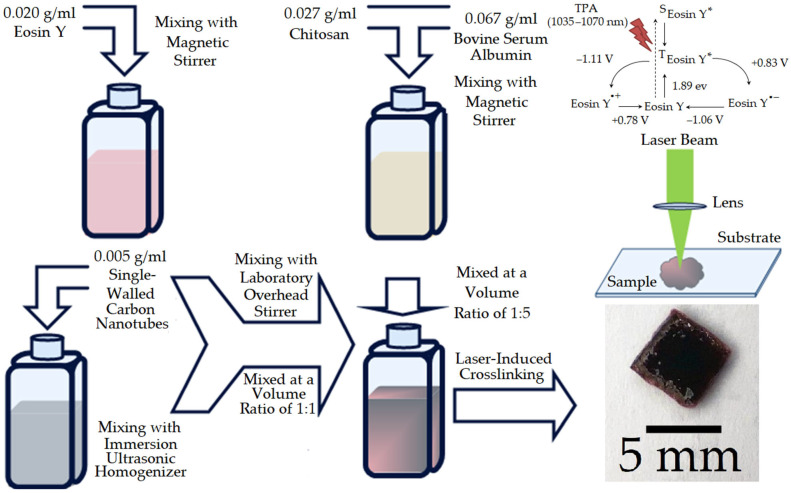
Manufacturing method.

Preparation of the gel containing CS (LLC ‘Bioprogress’, Losino-Petrovsky, Russia) and BSA (BioClot, Aidenbach, Germany) involves several steps (Figure 1), which ensure uniform distribution of the components throughout the entire volume. First, an aqueous solution of Eosin Y (Agat-Med, Moscow, Russia) was prepared using an Elmi MS-01 magnetic stirrer (ELMI, Riga, Latvia) to achieve a concentration of 0.020 g/mL. Simultaneously, a dispersed medium containing SWCNTs 0.005 g/mL produced by TUBALL^TM^ (OCSiAl, Moscow, Russia) was prepared. A stable dispersion was obtained using a Sonicator Q700 ultrasonic immersion homogenizer (Qsonica, Newtown, CT, USA) by processing for two hours at 20 kHz and 100 W power. Temperature-controlled cooling was applied to prevent boiling. BSA was gradually added to the CS dispersion medium in distilled water and stirred using a magnetic stirrer until a concentration of 0.027 g/mL was achieved.

**Scheme 1:**
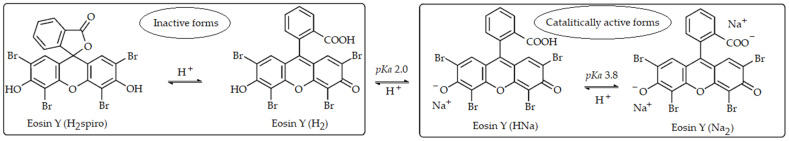
Acid–base equilibria of Eosin Y.

The polymer composite was formed using IR laser radiation at a wavelength of 1070 nm and a pulse duration of 200 ns. Two-photon absorption (TPA) in Eosin Y is similar to green light absorption (Figure 1), as illustrated in the typical photo-redox catalytic cycle diagram. Upon laser irradiation, intersystem crossing conversion occurs from the ground state to the lowest-energy triplet state (^T^Eosin Y*) [40,41]. In this case, the direct singlet-triplet transition is forbidden. However, electrons are excited to a higher singlet state (^S^Eosin Y*), from which they can rapidly relax to the lowest excited singlet state. Only after this, the excited electrons undergo intersystem crossing conversion to a highly reactive triplet state. The catalytic properties of Eosin Y depend on its oxidation-reduction potential: the oxidation potential ranges from −1.10 V to −1.06 V, while the reduction potential ranges from +0.78 V to +0.83 V. In aqueous solution, this dye exists in equilibrium among four different forms due to the presence of two relatively acidic protons (pKa 2.0 and 3.8 in water, Scheme 1). Only the monoanionic form of Eosin Y (HNa) and the dianionic form of Eosin Y (Na_2_) are catalytically active.

### 2.2. Evaluation of Nonlinear Optical Properties

**Figure 2:**
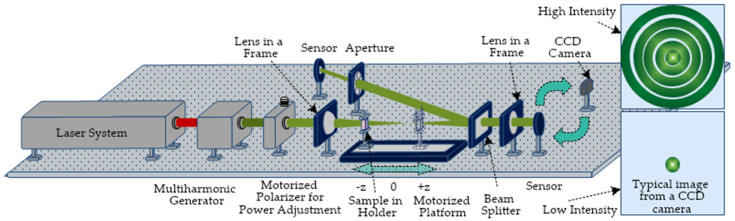
Scheme of the assembled stand.

All the prepared components were mixed to form the gel, which consisted of CS/BSA/Eosin Y/SWCNTs. Subsequent experiments were carried out on the laboratory bench (Figure 2). The regenerative femtosecond amplifier TETA-3-HE (AVESTA-PROJECT LLC, Troitsk, Moscow, Russia) generating pulses τ_f_ = 270 fs at the wavelength λ_f_ = 1035 nm and the HTTP MARK MOPA laser system (Bulat Design Bureau, Zelenograd, Russia) with pulse duration τ_n_ = 200 ns at the wavelength λ_n_ = 1070 nm were used as radiation sources with the same pulse repetition rate 30 kHz. A SP920s laser beam profilometer (Ophir Optronics Ltd., Jerusalem, Israel) equipped with BeamGage Standard software v6.19 was used to quantify the number of observed rings. The system provided a recording resolution of 1624 × 1224 pixels at a rate of up to 15 frames per second, covering a wavelength range of 190–1100 nm.

The Z-scan technique was employed to determine the nonlinear absorption cross-section σ and the nonlinear refractive index *n*_nr_. The experimental setup used a cuvette with an optical path length of *d* = 0.3 cm. The beam radii at the lens focus were measured as *w*_f_ = 23 μm for femtosecond pulses and *w*_n_ = 32 μm for nanosecond pulses. This difference in beam size arises from the different value of the initial laser beam diameters before focusing, despite using lenses with identical focal lengths in both cases. The molar concentration of Eosin Y in the solution was *C* = 1 mM.

Using open-aperture Z-scan measurements, the value of the nonlinear absorption cross-section was calculated using the following Equation (1):

(1) $$T_{norm}=\exp\left(\frac{2\lambda d\sigma N_{A}C\cdot 10^{-3}}{\tau h\nu w^{2}c\pi \sqrt{\pi }}\left(P_{th}-\frac{P_{0}z_{0}}{\left(z_{0}^{2}+z^{2}\right)}\right)\right)$$

where *N*_A_ is the Avogadro number; *P*th is threshold power for the observation of nonlinear effects; *h* is the Planck constant; *c* is the speed of light in vacuum; *z*_0_ is the Rayleigh length.

The nonlinear refractive index was determined using closed-aperture Z-scan measurements [42]. In this case, two measurements are performed. After acquiring the data, the closed-aperture Z-scan results were normalized by dividing them by the corresponding open-aperture Z-scan data. The theoretical curve was calculated using the following Equation:

(2) $$T_{norm}=1-\frac{16\pi n_{\mathrm{nr}}P_{0}L_{\mathrm{eff}}zz_{0}}{\tau \nu w^{2}\lambda \pi \sqrt{\pi }\left(z^{2}+9z_{0}^{2}\right)\left(z^{2}+z_{0}^{2}\right)}$$

where *L*_eff_ is effective length, which is determined by the equation *L*_eff_ = (1-exp (-α*d*))/α, where α is linear absorption coefficient.

Unlike in previous work [1], the experimental setup has been changed to enable real-time monitoring of the laser beam profile. This enhancement allows precise control of factors affecting beam shape uniformity, which is critical for achieving homogeneous composite polymerization. When the laser operates in frequency-modulated irradiation mode, a change in the spatial beam profile is observed after interaction with the resulting phase inhomogeneity. In this configuration, the nonlinear refractive index can be extracted from the experimental data by fixing the sample position relative to a closed-aperture lens and applying the Fresnel–Kirchhoff diffraction integral [43]. In the general case, the intensity *I* at the observation point *r*’ in the near-field diffraction zone (Fresnel zone) is generally expressed as:

(3) $$I\left(r^{\prime }\right)=\frac{1}{\lambda }\frac{2P_{0}}{w_{0}^{2}\pi }\int_{0}^{+\infty }\exp\left(-\frac{2r^{2}}{w^{2}}\right)\exp\left[i\left(-k\frac{{\left(r-r^{\prime }\right)}^{2}}{2R}\right)+n_{nr}\frac{2P_{0}}{w^{2}\pi }d\exp\left(-\frac{2r^{2}}{w^{2}}\right)\right]dr$$

where *R* is the radius of curvature of a wavefront and *k* is the wavenumber.

For a fixed sample position, the number of diffraction rings *N* was measured using a laser beam profilometer [30,44,45]. These measurements enabled determination of the nonlinear refractive index (*n*_nr_) through the following Equation:

(4) $$n_{\mathrm{nr}}=\frac{\lambda N\nu w^{2}\tau \pi \sqrt{\pi }}{4n_{0}L_{\mathrm{eff}}P_{0}}$$

where *n*_0_ is linear refractive index.

### 2.3. Spectral Studies

The optical density of the prepared gel was monitored at each preparation stage using a GENESYS 50 UV-Vis-Nir spectrophotometer (Thermo Fisher Scientific, Waltham, MA, USA). Absorption spectra were recorded across the 300–1100 nm wavelength range with a 2 nm spectral bandwidth. Raman and FTIR spectroscopy techniques were used to monitor the preservation of the initial components in the polymer composite, unlike in previous studies of gelatin-based hydrogels [1].

Raman spectroscopy was performed using a LabRAM HR Evolution HORIBA Scientific optical system (HORIBA, Palaiseau, France) with 633 nm excitation wavelength, 0.5 mW laser power, and the duration of 10 s.

FTIR spectra were acquired using Nicolet iS50 Thermo Scientific Fourier-transform (Thermo Fisher Scientific, Waltham, MA, USA) equipped with a diamond-ATR (attenuated total reflection) accessory, covering the spectral rang of 500–4000 cm^−1^ with resolution 1 cm^−1^.

### 2.4. Determination of Electrical Conductivity of the Formed Composite

The electrical conductivity was evaluated using an ST2258C four-point probe system with the van der Pauw method measurement system (Suzhou Jingge Electronics Co., Ltd., Suzhou, China). Square hydrogel samples (5 × 5 mm) were prepared to meet optimal measurement requirements.

### 2.5. In Vitro Experiments

The addition of any new component to the previously reported composition [1] may cause agglomeration and significant sediment formation in the hydrogel. Furthermore, it could affect biocompatibility, necessitating evaluation of these properties. This study utilizes Neuro 2A cells. The Neuro 2A is a mouse neuroblastoma cell line established in 1969 from a spontaneous tumor in a line A albino mouse. This line is a convenient model of neuronal cells, widely used by researchers in studies of neuronal development and differentiation [46]. The cells express microtubule proteins that contribute to the contractile system mediating axoplasmic flow in nerve cells [47]. Neuro 2A cell line requires relatively simple culture conditions, making it accessible for most research laboratories. Its applications span investigations neuronal development, differentiation, neurotoxicity, cell signaling pathways, and axon growth. The cells are used to develop and test various therapeutic agents, including those with nanoparticles in their composition [48].

In vitro studies of Neuro 2A cell line allow us to evaluate the applicability of the developed composite as neurointerface material, which supports neuronal growth.

Neuro 2A cells were purchased from the National Research Center for Epidemiology and Microbiology of the Ministry of Health of the Russian Federation for biocompatibility assessment studies. Prior to experiments, samples were sterilized with ultraviolet light and washed with cell medium. Cells were cultured in DMEM and 90% culture medium supplemented with 10% calf fetal serum. Clean cover glass was used as the control sample. The cell seeding dose was 2.4 × 10^5^ cells/mL, cell concentration was counted with an automatic counter Scepter Millipore (Merck KGaA, Darmstadt, Germany). The cells were incubated in a thermostat with 5% CO_2_ atmosphere at 37 °C for 72 h. At the end of the cultivation, MTT test was performed according to the standard method for assessing cell viability [49]. Optical density was recorded using a microplate photocalorimeter Immunochem-2100 (High Technology Inc., North Attleboro, MA, USA) at a wavelength of 492 nm. Cells were stained for fluorescence visualization with Hoechst 33,342 dye (Life Technologies, New York, NY, USA) at 10 mg/mL. Cell morphology was analyzed using fluorescence microscopy images, obtained with microscope Olympus FV3000 (Olympus Corporation, Tokyo, Japan).

## 3. Results

### 3.1. Nonlinear Optical Properties

#### 3.1.1. Experiments

**Figure 3:**
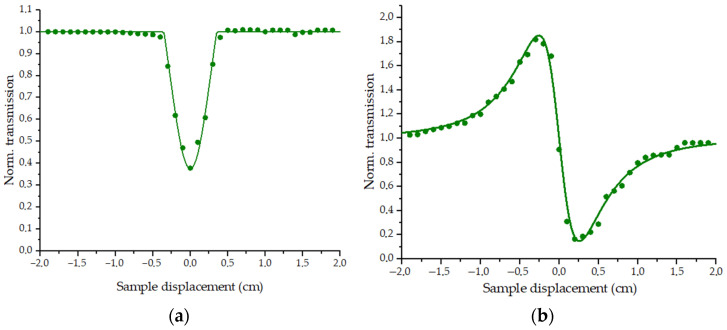
Z-scan results of CS/BSA/Eosin Y/SWCNTs at nanosecond laser pulses: (**a**) open aperture; (**b**) closed aperture.

**Figure 4:**
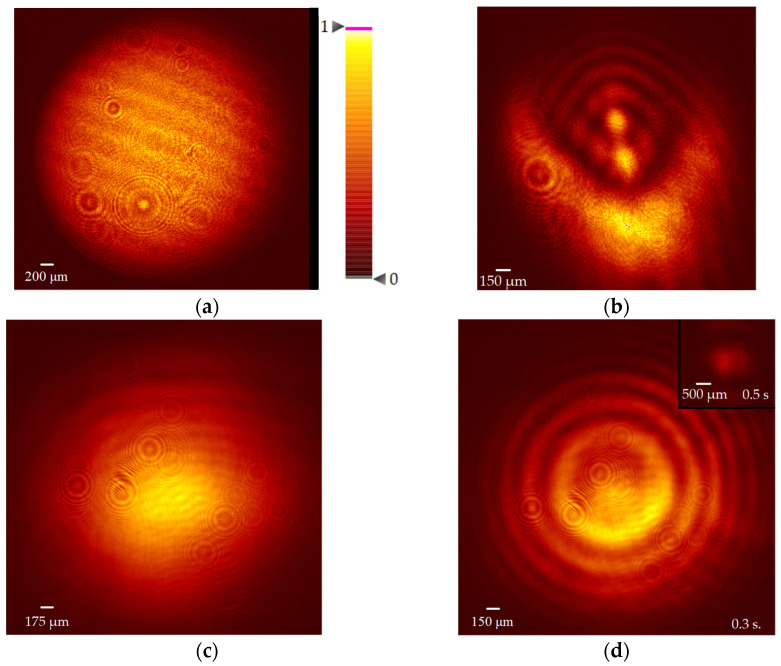
Beam profile when irradiating a sample with laser pulses: nanosecond duration (**a**) far from focus and (**b**) in focus; femtosecond duration (**c**) far from focus and (**d**) in focus.

As we have previously demonstrated [1], even varying the gelatin content—which itself shows no laser radiation absorption—affects the material’s nonlinear optical properties through increased scattering caused by extended optical path length during multiple scattering events. As a consequence, an increase in the nonlinear absorption cross-section value is observed during two-photon polymerization. Therefore, in order to maintain a sufficient nonlinear response, the concentration of CS in the initial composition was selected. A very important result of this work is the determination of the required nonlinear absorption cross-section value, which was more than 500 GM for the CS-based composition. This nonlinear absorption cross-section value was achieved with stronger changes in the nonlinear refractive index-twice as much as in the case of the gelatin-based composition (without CS). Stronger changes in refraction in the CS-based composition are confirmed by the obtained new effect of phase self-modulation with the appearance of diffraction rings. This effect was first observed in this work when femtosecond and nanosecond radiation was applied to a dispersed medium based on CS/BSA/Eosin Y/SWCNTs. The values of the nonlinear absorption cross-section σ = 515 GM and the threshold power *P*th = 240 mW were determined using the data of open aperture Z-scan (Figure 3a) and Equation (1). The nonlinear refractive index, determined via closed-aperture Z-scan (Figure 3b) with Equation (2), was *n*_nr_ = (9.5 ± 1.0) 10^−11^ cm^2^/W. The number of rings was also estimated using the data of a fixed sample location method. The shape of the original beam (Figure 4a) takes the form corresponding to diffraction rings (Figure 4b) when the sample is located in the focus of the lens. The value of the nonlinear refractive index according to Equation (4) was *n*_nr_ = (10.0 ± 1.0)·10^−11^ cm^2^/W, which is consistent with the value previously determined by the closed aperture Z-scan.

An analogous experimental procedure was conducted using femtosecond pulses. When the sample was positioned at the lens focal point, the initial Gaussian beam profile (Figure 4c) transformed into a distinct diffraction ring pattern (Figure 4d). The value of the nonlinear refractive index *n*_nr_ = (1.2 ± 0.5)·10^−15^ cm^2^/W was determined using Equation (4).

The inset in Figure 4d displays the pattern observed 0.5 s after initiating laser exposure. The diffraction rings emerged approximately 0.3 s following irradiation onset. Polymer composite formation altered the diffraction pattern, manifesting as a sharp decrease in transmission and consequent reduction in transmitted intensity. All images in Figure 4 maintain identical scaling, normalized to maximum intensity when the sample was positioned far from the focal plane.

**Figure 5:**
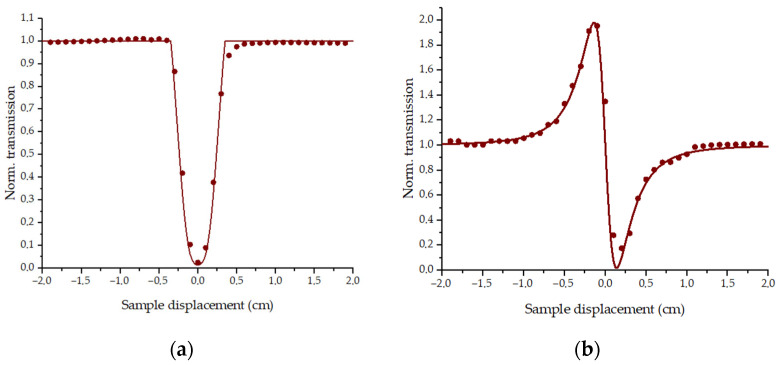
Z-scan results of CS/BSA/Eosin Y/SWCNTs at femtosecond laser pulses: (**a**) open aperture; (**b**) closed aperture.

The study of a new effect of phase self-modulation in the case of excitation of a CS-based composition by femtosecond laser radiation allows for control over the process of two-photon polymerization. It is important to determine the time of completion of two-photon polymerization to avoid excessive exposure of biopolymers to radiation. This is necessary to reduce heating of the area outside the focusing zone, which allows for control over the size of the formed voxel. Open aperture Z-scan measurements with femtosecond laser pulses (Figure 5a) and Equation (1) yielded a nonlinear absorption cross-section of σ = 19 GM at a threshold power Pth = 6 mW. Closed-aperture Z-scan analysis using Equation (2) determined the nonlinear refractive index as *n*_nr_ = (1.2 ± 0.5) × 10^−11^ cm^2^/W. The obtained value agrees, within experimental error, with the value determined using Equation (4).

The obtained data demonstrate a strong correlation between the nonlinear refractive index and the nonlinear absorption cross-section. The extended optical path length within the sample due to scattering enhances the probability of TPA. At low values of the nonlinear refractive index, the nonlinear absorption cross-section primarily reflects TPA, which is fluence-dependent, while when values are significant, it exhibits reduced influence in the nanosecond regime. Thus, the obtained data confirm the importance of the identified effect of phase self-modulation capable of enhancing nonlinear absorption.

#### 3.1.2. Theoretical Research

**Figure 6:**
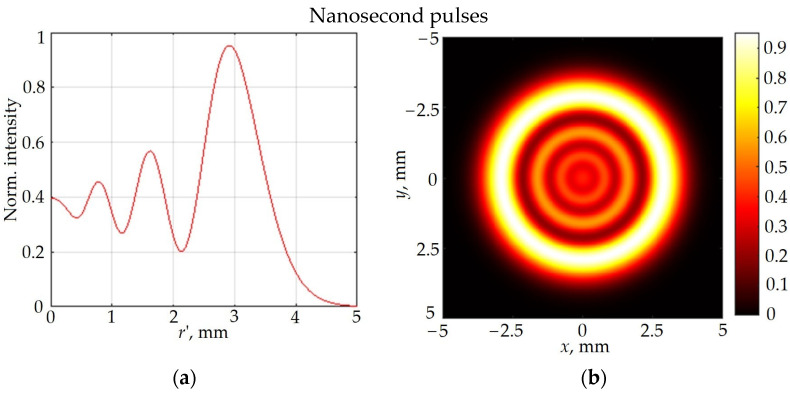

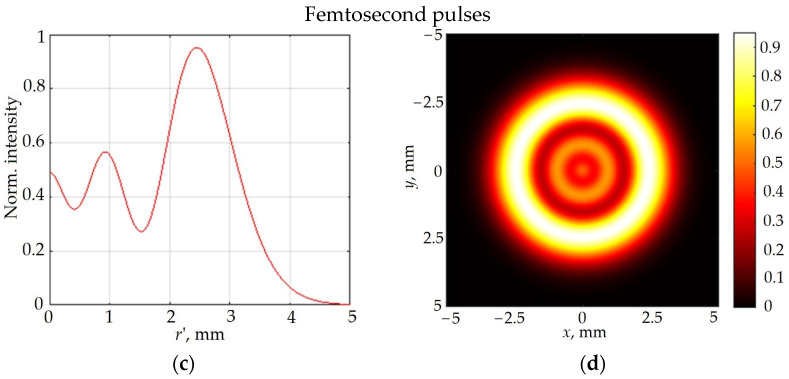
Spatial beam profiles after interaction with the sample: radial (**a**,**c**) and two-dimensional (**b**,**d**) patterns for radiation with nano- and femtosecond pulse durations in the absence of thermal convection.

Using the measured nonlinear refractive index values under the action of laser radiation with nano- and femtosecond laser irradiation, we determined diffraction patterns in the hydrogel prior to thermal motion initiation. The radial profiles of the diffraction pattern (Figure 6a,c) were calculated using Equation (3) with the sample positioned at the lens focal point. At a recording plane distance of 5 cm, beam radii were 32 and 23 μm for nano- and femtosecond pulses, respectively. From these profiles, we reconstructed two-dimensional spatial beam distributions after hydrogel transmission. The calculated parameters accurately describe the beam profiles observed both during initial irradiation and prior to diffraction pattern disruption caused by solid polymer composite formation within the hydrogel matrix (Figure 6b,d).

Figure 6 demonstrates that higher nonlinear refractive index values and greater beam waist radius during nanosecond pulses irradiation result in more numerous diffraction rings despite comparable beam expansion with a similar expansion. The negligible thermal effects during initial irradiation produce a uniform refractive index gradient within the irradiated zone. In turn, this generates a symmetric diffraction pattern in the detection plane. The homogeneous hydrogel properties across the irradiated region enable the formation of symmetrically shaped solid polymer composites, permitting reduced voxel dimensions in this fabrication approach.

### 3.2. Spectral Analysis

**Figure 7:**
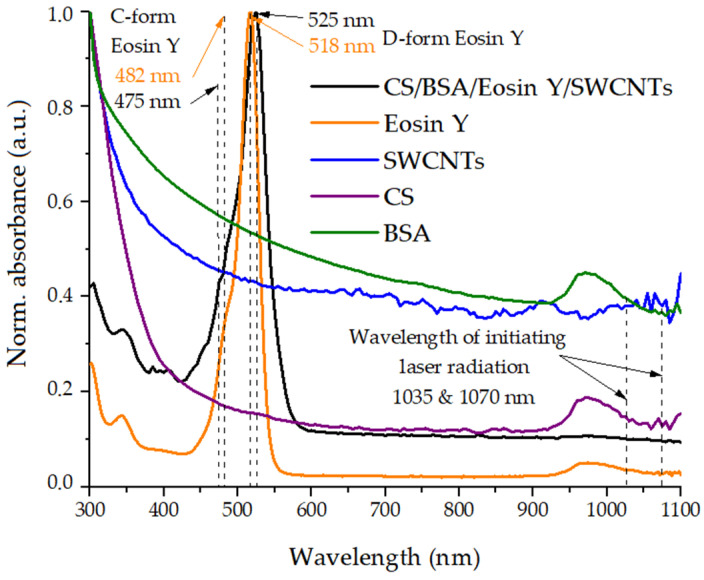
Absorption spectrum of gel CS/BSA/Eosin Y/SWCNTs.

Figure 7 presents the absorption spectra of the prepared hydrogel and constituent components, with peak absorbance values normalized to unity. A transmission window emerges for 1035–1070 nm laser radiation, confirming negligible one-photon absorption. In the near IR region, water displays bands with a maximum at 980 nm and shorter-wavelength weak bands [50]. In case of components including CS, BSA, and SWCNTs, UV spectral maxima suggest possible three-photon absorption under intense irradiation [51]. For this reason, the selected power values were limited by the significant manifestation of nonlinearity in the obtained Z-scan data. Within the TPA range (500–550 nm), Eosin Y displays an absorption maximum for both 1035 and 1070 nm excitation. In the CS/BSA/Eosin Y/SWCNTs hydrogel, this peak undergoes a bathochromic shift from 518 to 525 nm upon binding to amino acid residues of BSA [52] and SWCNTs [53]. Concurrently, a change in the ratio between the C-form and D-form bands is observed, which indicates minor pH variations. Our studies confirm that CS, similar to gelatin [1], exhibits negligible laser radiation absorption at both the employed laser wavelengths and within the TPA spectral window.

**Figure 8:**
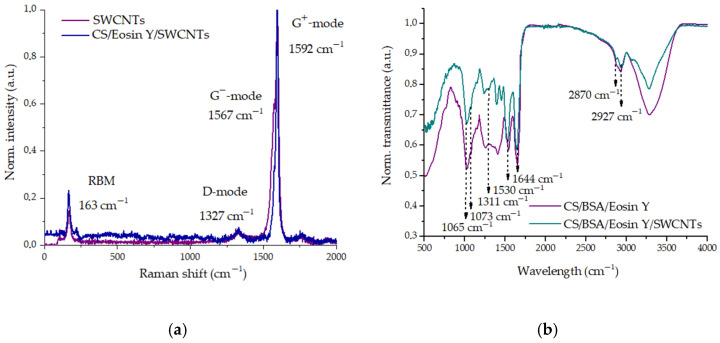
Spectroscopy data: Raman spectrum of SWCNTs and CS/Eosin Y/SWCNTs (**a**); FTIR spectrum of CS/BSA/Eosin Y/SWCNTs and CS/BSA/Eosin Y (**b**).

Figure 8a presents the Raman spectroscopy results. For SWCNT (Figure 8a), the radial breathing mode (RBM) appeared at 164 cm^−1^. Since the RBM frequency is inversely proportional to nanotube diameter [54], this measurement yielded a diameter of 1.5 ± 0.2 nm. In our experiments, the response represented a superposition of multiple vibrational modes, since the excitation photon energy of 1.96 eV was in resonance with several chiral modes [55]. The splitting of the tangential G band (sp2 component) into high-intensity G+ and low-intensity G- confirms the semiconducting nature of the nanotubes. The shape characteristic lineshape of the split G band indicates the inclusion of significantly bundled (aggregated) SWCNT in the sample. The 25 cm^−1^ splitting between these bands additionally enables determination of the SWCNT diameter as 1.5 ± 0.2 nm [54]. The ratio of the integrated intensities of the D mode (sp_3_ component) to the G+ mode intensity is commonly used to estimate the sp_3_ density. The SWCNT sample exhibits an I_D_/I_G+_ ratio of 0.143, indicating the presence of defects in the binding to COOH groups. When interacting with other composite components CS/Eosin Y/SWCNTs, this ratio remains unchanged. However, the Raman signal becomes undetectable upon BSA incorporation. The CS/BSA/Eosin Y/SWCNTs sample exhibited strong fluorescence at the selected excitation wavelength, which was attributed mainly to BSA autofluorescence (confirming the structural integrity of its aromatic amino acids) and the contribution of Eosin Y [56,57]. This fluorescence background significantly interfered with data acquisition. Figure 8b presents the FTIR spectrum of the obtained CS/BSA/Eosin Y/SWCNTs samples. The broad absorption band at 3000–3500 cm^−1^ indicates the OH stretching vibrations of the carboxyl or hydroxyl groups of eosin Y [58], BSA, and CS [59,60]. Symmetric and antisymmetric C-H stretching were observed at 2870 and 2927 cm^−1^, respectively [53]. The interaction of eosin Y and SWCNT led to small red shifts. The structure of CS is characterized by the presence of bands at 1644, 1530, and 1311 cm^−1^ (Amide I, II, and III, respectively) in the FTIR spectrum [29]. The vibration bands of the C–O valence vibrations in CS were observed at 1065 and 1073 cm^−1^ [60]. A strong NH_3+_ band at 1644 cm^−1^ indicated the presence of interactions between the components. For nanodiamonds, only diamond crystal ATR spectroscopy proves applicable, though this technique cannot directly measure carbon atom vibrations. However, it successfully detects absorption from nanodiamond surface functional groups [61]. Figure 8b demonstrates that increasing the SWCNTs concentration enhances the signal across nearly the entire spectral range. CS and BSA, being predominantly cations, form ionic interactions and hydrogen bonds with the anionic active forms of Eosin Y. SWCNTs stabilize through weak van der Waals interactions, acting as secondary stabilization mechanisms. The laser radiation cessation triggers a rapid cooling-like process that preserves bonds and prevents defects.

### 3.3. Electrical Conductivity of the Formed Composite

The polymer composite was formed with a laser spot movement speed of 240 mm/s using a scanning system. With such exposure parameters, 14 repetitions were performed along one line, which allowed constant irradiation to be provided for 0.3 s with a line length of 5 mm. This duration proved sufficient for diffraction rings formation, as previously demonstrated (Figure 4d). The radiation intensity was maintained constant, which should be at least 3 MW/cm^2^, as determined by Z-scan measurements. The two-photon-polymerized SWCNTs-based composite exhibits a specific conductivity of about 20 mS×cm^−1^, measured via the four-point probe van der Pauw method. Replacing gelatin [1] with CS did not compromise electrical conductivity. During stimulation, the physiological electrical potential remains within about 100 mV, while the required bioactive current ranges from 0.6 to 400 μA. For neural interface applications in damaged neural network repair, a specific conductivity above 1.0 mS×cm^−1^ suffices to guide neurite outgrowth during regeneration and enable functional electrical stimulation.

### 3.4. Biocompatibility of Formed Composite Samples

**Figure 9:**
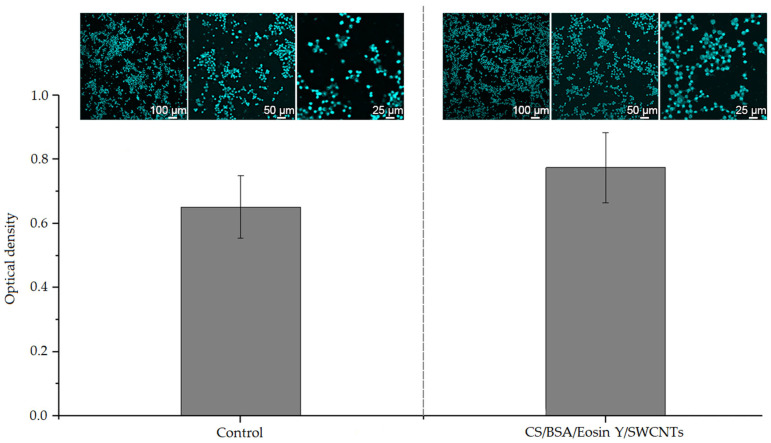
Cell morphology and biocompatibility studies results for sample CS/BSA/Eosin Y/SWCNTs and control samples with different magnification after 72 h of cultivation.

At the final research stage, biocompatibility properties were evaluated according to the study protocol. Figure 9 presents the MTT assay results. It was found that the studied nanocomposites promote Neuro 2A cell line proliferation, showing a 16% increase in cell count compared to the control sample. The fluorescence microscopy data corroborate the MTT test (Figure 9). A greater number of cells are visible on the surface of the CS/BSA/Eosin Y/SWCNTs sample, and they are distributed over the surface more evenly. Cell morphology on the experimental sample matches the control; thus, we can conclude that there is no toxic effect from the experimental sample and it has a positive effect on cell proliferation. In the case of gelatin previously used [1], the amount was within the permissible values, as the current results of the work show; in the case of CS, their amount exceeded the control results by 16%. In the present study, the planting dose of Neuro 2A cells is somewhat lower, and the cells showed a greater tendency to aggregate into agglomerates. At the same time, for the experimental samples, which is more favorable for the effective transmission of signals between neurons [62], the cells become larger, with a parallel increase in the cellular perimeter, and due to the appearance of a developed network of processes, they have a non-spherical shape, which is characteristic of neuronal differentiation and active proliferative status [63] (Figure 9).

## 4. Discussion

Laser radiation induces concentric diffraction rings via self-phase modulation, which then experience thermal distortion [64,65]. Comparing the nonlinear refractive indices between ultrafast pulsed and a continuous-wave laser radiation revealed significant differences [66]. This observed discrepancy reflects coherent light scattering phenomena, where continuous-wave irradiation exhibits lower intensity due to pronounced thermal effects compared to ultrafast laser excitation. Our study specifically compared ultrafast femtosecond and fast nanosecond irradiation modes. When selecting power parameters, we accounted for potential multiphoton effects that could contribute to nonlinear absorption, was taken into account [51]. The nonlinear refractive index measured lower for pulses with a duration of τ_f_ = 270 fs compared to τ_n_ = 200 ns at identical repetition rate, suggesting thermal effect influence [67,68,69,70]. Nonlinear optical effects became observable at substantially lower values of laser radiation power for femtosecond pulses (35 mW) compared to nanosecond pulses (570 mW), consistent with enhanced thermal effects in the nanosecond regime [71]. Consequently, ultrafast pulses absorb less energy than nanosecond pulses, minimizing diffraction ring distortion while retaining sufficient energy for hydrogel volume polymerization. In this case, the nonlinear refraction is caused by the viscosity and polarity of the solvent [72,73]. In our case, the dispersed medium is multicomponent, with each of the components affects the dynamic viscosity. Consequently, these parameter values sufficiently influence the diffraction ring pattern formation through significant scattering, increasing absorption capacity by extending the optical path length within the sample [1,29]. Thus, the nonlinear absorption cross-section was 19 GM for femtosecond pulses and 515 GM for nanosecond pulses, indicating a strong influence of scattering, which can increase the probability of two-photon absorption. This can be observed by the change in the nonlinear refractive index, which varies from 1.2 × 10^−15^ cm^2^/W for ultrafast pulses to 9.5 × 10^−11^ cm^2^/W for the nanosecond pulses.

## 5. Conclusions

Pain suppression can be achieved by neurostimulation therapy. This approach offers several advantages, particularly the avoidance of constant drug administration, which may prove ineffective due to interindividual variability. The application of neurostimulators is limited by scar tissue formation, which impairs feedback and can lead to complete signal transmission loss following neuronal death in the interface area. The study presents a polymer composite with sufficient specific conductivity of about 20 mS×cm^−1^, which simultaneously demonstrates sufficient biocompatibility with the Neuro 2A cell, as confirmed by three-day MTT assays. The original aqueous gel comprises biopolymers (CS and BSA), SWCNTs, and Eosin Y. Samples of the required shape were fabricated from this material via two-photon polymerization. Conventionally, this process requires complex femtosecond laser systems. The paper presents the studies of the nonlinear absorption cross-section, demonstrating scattering’s strong influence on this parameter. Thus, for nanosecond pulses, the nonlinear refractive index reaches 9.5 × 10^−11^ cm^2^/W, exceeding that of femtosecond pulses (1.2 × 10^−15^ cm^2^ /W). The values obtained with Z-scan method show good correlation with those determinations from diffraction rings pattern analysis. Consequently, the coherent scattering of light in case of nanosecond pulses is stronger, which leads to an increase in the optical path length inside the sample. The obtained data confirm the significance of the revealed self-phase modulation effect, which enhances nonlinear absorption. The nonlinear absorption cross-section reached 515 GM for nanosecond pulses, exceeding that of femtosecond pulses (19 GM). However, nonlinear effects require significantly higher threshold laser power, which was 240 mW and 6 mW, respectively. In this case, the CS and BSA, containing cationic groups, form ionic interactions with Eosin Y anions, accompanied by hydrogen bond formation. SWCNTs additionally interconnect via weak van der Waals forces, acting as secondary stabilization mechanisms, as verified by spectroscopy analysis. The self-phase modulation study targets achieving uniform hydrogel property distribution within the irradiation area and producing symmetrically shaped polymer composites. This approach enables reduction of three-dimensional voxels dimensions during polymer composite fabrication. The results demonstrate that femtosecond laser irradiation produces symmetrical diffraction rings pattern, indicating more uniform energy deposition. The low threshold exposure values also indicate potential for efficient laser power utilization in the case of ultrashort pulses. The two-photon polymerization process requires a specific exposure time of 0.5 s when using laser radiation with 270 fs pulse duration at a 30 kHz repetition rate. Composite formation requires establishing bonds between components. This process demands more time than a single pulse duration, necessitating laser irradiation with a sufficient pulse repetition frequency of 30 kHz to maintain the required process activity.

## Data Availability

Data underlying the results presented in this paper are not publicly available at this time but may be obtained from the corresponding authors upon reasonable request.

## Abbreviations

The following abbreviations are used in this manuscript:

BSA	Bovine serum albumin
SWCNTs	Single wall carbon nanotubes
FDA	Food and Drug Administration
CS	Chitosan
Eosin-Y	Eosin Yellow
TPA	Two-photon absorption
FTIR	Fourier transform infrared spectroscopy
ATR	Attenuated total reflection
SEM	Scanning electron microscope
MTT	Colorimetric assay for assessing cell metabolic activity
UV-Vis-Nir	Ultraviolet-Visible-Near Infrared
C-form	Cationic forms
D-form	Dianionic form
RBM	Radial breathing mode
G mode	Mode of graphite
D mode	Mode of diamond
